# The Role of Fibroblast Growth Factor (FGF) Signaling in Tissue Repair and Regeneration

**DOI:** 10.3390/cells10113242

**Published:** 2021-11-19

**Authors:** Mariya Farooq, Abdul Waheed Khan, Moon Suk Kim, Sangdun Choi

**Affiliations:** 1 Department of Molecular Science and Technology, Ajou University, Suwon 16499, Korea; mariyafarooq03@gmail.com (M.F.); waheedmarwat31@gmail.com (A.W.K.); moonskim@ajou.ac.kr (M.S.K.); 2 S&K Therapeutics, Ajou University Campus Plaza 418, 199 Worldcup-ro, Yeongtong-gu, Suwon 16502, Korea

**Keywords:** FGF, FGFR, tissue repair, tissue regeneration, cell proliferation, cell differentiation

## Abstract

Fibroblast growth factors (FGFs) are a large family of secretory molecules that act through tyrosine kinase receptors known as FGF receptors. They play crucial roles in a wide variety of cellular functions, including cell proliferation, survival, metabolism, morphogenesis, and differentiation, as well as in tissue repair and regeneration. The signaling pathways regulated by FGFs include RAS/mitogen-activated protein kinase (MAPK), phosphatidylinositol-4,5-bisphosphate 3-kinase (PI3K)–protein kinase B (AKT), phospholipase C gamma (PLCγ), and signal transducer and activator of transcription (STAT). To date, 22 FGFs have been discovered, involved in different functions in the body. Several FGFs directly or indirectly interfere with repair during tissue regeneration, in addition to their critical functions in the maintenance of pluripotency and dedifferentiation of stem cells. In this review, we summarize the roles of FGFs in diverse cellular processes and shed light on the importance of FGF signaling in mechanisms of tissue repair and regeneration.

## 1. Introduction

FGFs are a family of cell-signaling proteins produced by different types of cells. The first members of the fibroblast growth factor (FGF) family (FGF1 and FGF2) were discovered in the 1970s and were initially named acidic and basic FGF, respectively [1]. FGFs can affect the proliferation of multiple cells, including endothelial cells, neurons, chondrocytes, smooth-muscle cells, melanocytes, and adipocytes [2]. They are involved in a variety of biological processes, including cellular proliferation, survival, metabolism, morphogenesis, differentiation, embryonic development, angiogenesis, tissue repair, and regeneration [3]. FGFs trigger signaling by interacting with tyrosine kinase receptors, known as FGF receptors (FGFRs). The FGFR family of receptor tyrosine kinases, discovered in the 1970s, consists of four transmembrane receptors: FGFR1–4 [4]. Each of them contains three extracellular immunoglobulin-like binding domains, followed by a transmembrane domain and an intracellular domain constituting a two-part tyrosine kinase [5]. For signal transduction by most FGFs, their binding to coreceptors, either Klotho proteins or heparan sulfate (HS) proteoglycans, on the target cell surface is necessary too [6]. 

At present, 22 FGFs (i.e., ligands) are known, of which 18 interact with and induce the dimerization of FGFRs. Upon stimulation, these receptors activate downstream signaling cascades through their intracellular domains. In the absence of ligand, the inactive configuration of an FGFR kinase is allosterically autoinhibited by its hinge region and activation loop. In the presence of an extracellular ligand, FGFR dimerizes and autophosphorylates. Thus, it launches several signaling pathways, e.g., phospholipase Cγ (PLCγ), PI3K–AKT, and RAS–MAPK cascades [5]. FGFs are expressed in a variety of tissues and show differential expression according to metabolic requirements. Multiple isoforms of FGFs and FGFRs exist because of alternative splicing and alternative translational initiation sites. The expression of FGFs is also regulated at the epigenetic and post-translational levels, including phosphorylation, glycosylation, ubiquitination, and cellular trafficking [7]. 

**Figure 1:**
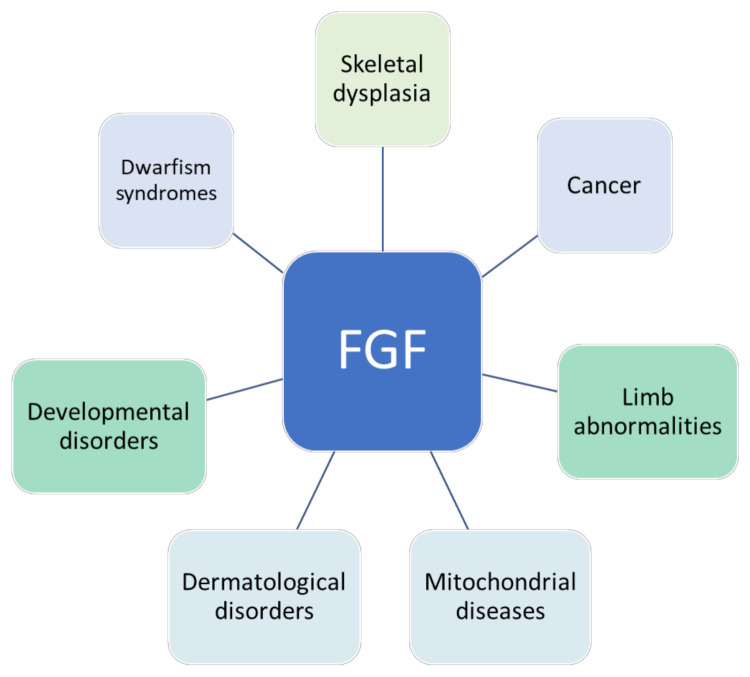
Diseases associated with FGFs dysregulations. Abnormalities in FGF signaling can lead to dwarfism syndrome [19], skeletal dysplasia [20], limb abnormalities [21], mitochondrial diseases [9], cancers, skin diseases [22], and developmental disorders [11].

Dysregulated FGF signaling contributes to many diseases, including multiple cancer types, skeletal system defects, developmental diseases, chondrodysplasia, corneal neovascularization, and X-linked hypophosphatemic rickets [8], as shown in Figure 1. FGF21 has been proposed to be a potential biomarker for mitochondrial diseases [9]. Aberrant activity of different types of FGF can result in developmental disorders [10,11]. FGF23 is expressed at a high level in bone cells, particularly in osteoclasts. The expression of FGF23 has been found to be upregulated in bone disorders and chronic kidney disease as well [12,13,14,15]. Cancer-associated fibroblasts are activated fibroblasts that serve as a key component of the tumor microenvironment [16]. They trigger the MEK–extracellular signal-regulated kinase (ERK) pathway and modulate MMP7 through FGFR4 to stimulate cancer cell growth and angiogenesis [17]. Moreover, an FGF is hypothesized to contribute to the development of chemoresistance in cancer cells [18]. Therefore, it is clear that aberrant FGF activity can cause several cancers because of ligand or receptor overexpression or somatic mutations in *FGFR* genes. 

Angiogenesis is one of the key regulators of wound healing. Blood vessels transport nutrients, oxygen, and immune cells which speed up the process of the wound healing process. The presence of mature blood vessels is very helpful for boosting tissue repair. Both acidic and basic FGF (bFGF) are involved in angiogenesis in particular ways. bFGF has been found at a high level in acute wounds and promotes wound healing and tissue remodeling [8]. It has also been demonstrated by in vivo studies that treating wounds with FGF speeds up the process of tissue repair. FGF can also be administered through biomaterials and different polymers for tissue repair and regeneration [23,24,25]. Consequently, the role of FGFs in the healing of wounded tissues appears to be indispensable, and multiple research articles have confirmed that FGFs play a part in tissue repair and regeneration and highlight the interplay between FGFs and other critical signaling molecules. These new insights into the functions of FGFs in tissue repair and regeneration are summarized in this review.

## 2. Fibroblast Growth Factor Family

Growth factor effects were first identified in 1939 by Trowell and Willmer, who observed mitogenic activity in the saline extract of a chick [26]. In 1973, Armelin reported that pituitary extracts are capable of stimulating the growth of 3T3 cells [27]. In 1974, Denis Gospodarowicz demonstrated that the active ingredient of the pituitary extract is an FGF [28]. In 1989, Burrus et al. coined another name for FGF—heparin-binding growth factor—and suggested that the active ingredient obtained by Trowell and Willmer (1939) in their experiment on the saline extract from the chick was FGF, along with other growth factors [29]. With the identification of heparin as a cofactor, the purification of FGF1 and FGF2 became possible. The first FGFR was discovered soon after the identification of the role of heparin in FGF transport between cells. It has also been found that some FGFs do not bind to HS. Rather, another protein, Klotho protein, had a higher affinity for this specific type of FGF. Mechanisms underlying the regulation of FGF activity include HS binding, N-terminal alternative splicing, homodimerization, and site-specific proteolytic cleavage [30]. HS binding not only determines the mode of action of an FGF but also influences the activity of other paracrine ligands from the same subfamily. The HS glycosaminoglycan binding site consists of a β1–β2 loop and is located inside the FGF core [31]. 

Members of this family are 150–300 amino acid residues in length and consist of a core region composed of six identical subunits of 120 amino acid residues. The core subunit is highly conserved, with 30–60% homology between FGFs. FGFs usually have a mass of 18 kDa; however, it may be as low as 7 kDa for FGF1 and as high as 389 kDa in the case of FGF5 [2]. In mammals, the FGF family consists of 22 members divided into seven subfamilies, as presented in Table 1.

**Figure 2:**
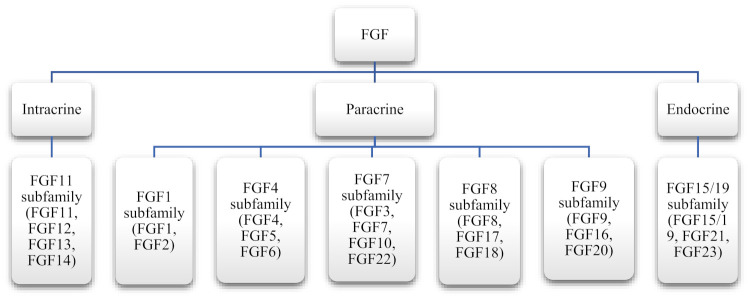
FGFs’ classification according to their mode of actions.

The subfamilies are classified based on sequence and structure. Initially, during evolution, the ancestors of FGF subfamilies arose, which then gave rise to three to four members later. Their exact evolutionary history is unknown [32]. On the basis of the mode of action, the seven subfamilies of FGF are divided into three categories: autocrine, paracrine, and endocrine. Out of the seven subfamilies of FGF, six belong to the intracrine/paracrine mode of action, while one (FGF19) belongs to the endocrine mode of action (Figure 2) [33]. Intracrine FGFs, as the name suggests, are intracellular molecules and are not secreted extracellularly. However, paracrine FGFs are extracellular proteins and bind to the FGFRs through heparin or heparan sulfate. Endocrine FGFs interact with FGFR with the help of another cofactor, Klotho proteins. Their binding affinity with heparan sulfate is relatively lower [34]. 

## 3. Receptor Activation Mechanism

**Figure 3:**
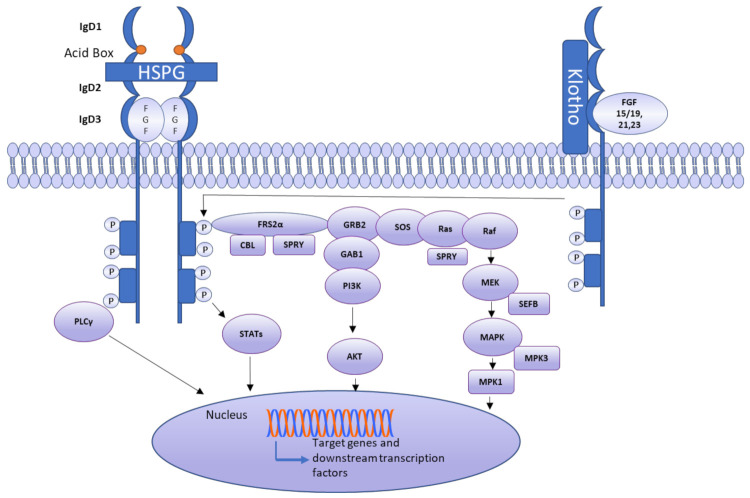
FGF signaling pathway. When a ligand interacts with an FGFR, it causes conformational changes in the receptor, leading to receptor dimerization and thus FGFR activation. When FGFR is activated, FRS2a is phosphorylated, causing FGFR substrate to attach to the Src Homology 2 (SH2) domain-containing adaptor growth factor receptor-bound protein 2 (GRB2). GRB2 binds SOS, GRB2-associated-binding protein 1 (GAB1), and Casitas B-cell lymphoma (Cbl) protein via the SH3 domain and stimulates Ras/Raf and mitogen-activated protein kinases (MAPK), including ERK, p38, and JNK. In addition to MAP kinases, STAT, PI3K, and PLCγ pathway is also activated.

An FGFR is comprised of three extracellular immunoglobulin-like domains (D1–D3), a single-pass transmembrane domain, and a cytoplasmic tyrosine-kinase domain. A linker, called the acid box, is located between D1 and D2 (Figure 3). The acid box, as its name indicates, is acidic and has a serine-rich sequence, which is a hallmark of FGFRs [31]. The acid box and D1 domain are thought to function in receptor autoinhibition, whereas the D2–D3 domains of an FGFR are essential for ligand binding and specificity. Multiple FGFR isoforms have been documented. Exon skipping and alternative splicing are the primary mechanisms reported to generate isoforms [31]. Two isoforms of FGFR1–3 are generated through alternative splicing and are often termed IIIb and IIIc. Epithelial cells express FGFR IIIb, whereas FGFR IIIc is usually expressed by mesenchymal cells. The *FGFR4* gene does not undergo alternative splicing; therefore, it does not have isoforms [35]. FGFs 11–14 do not interact with FGFRs because these ligands are intracellular proteins [36]. The known interactions of FGFs with FGFRs are listed in Table 2.

Ligand binding induces conformational changes in FGFRs, thus causing receptor dimerization and activation. Upon this activation, the phosphorylation of FGFR substrate a (FRS2a) occurs, leading to the binding of FRS2a to Src homology 2 (SH2) domain-containing adaptor growth factor receptor-bound protein 2 (GRB2). Next, GRB2 binds through its SH3 domain to SOS, GRB2-associated-binding protein 1 (GAB1), and the Casitas B-cell lymphoma (CBL) protein, subsequently stimulating Ras, Raf, and mitogen-activated protein kinases (MAPKs), including ERK, p38, and JNK. The phosphoinositol-3 kinase (PI3K) pathway is also launched by an FGFR, activating AKT. FGFRs stimulate and phosphorylate PLCγ as well, as shown in Figure 3 [55]. As a consequence of FGFR engagement, the phosphorylation of signal transducer and activator of transcription (STAT) also occurs. Multiple other mechanisms activate FGFRs, including mutation, translocation, or gene amplification. Increased levels of a circulating FGF can also result in FGFR triggering [56]. Although evidence is lacking, some mechanisms of negative feedback regulation of FGF signaling have been proposed. Members of a similar expression to the FGF (SEF) family can inhibit the signaling of an FGFR by interacting with its cytoplasmic domain. It has been suggested that FGFRL1 (atypical FGFR, also known as FGFR5) may serve as a ligand trap, dimerize with other transmembrane FGFRs, and prevent autophosphorylation or raise the turnover rate of other FGFRs [57]. 

HSs are unbranched carbohydrate chains composed of disaccharide repeats of N-substituted glucosamine and glucuronic acid that are frequently sulfated at numerous positions on their sugar residues. HSs can be in the form of unconjugated chains or be a part of HS proteoglycans by their conjugation to amino acids. HSs are involved in cellular growth, development, homeostasis, adhesion, and anticoagulation [58,59]. The roles of HS and heparan sulfatases in cancers and viral diseases have been investigated extensively. It has been reported that HS facilitates virus entry and can serve as a receptor for viral attachment [60,61]. HS abnormalities have been implicated in solid tumors and hematological cancers because it promotes oncogenesis by enhancing tumor cell proliferation, metastasis, and dedifferentiation, as well as angiogenesis [62]. The expression of HS is controlled by modifications and an enzyme responsible for its synthesis. Abnormal expression of HS-modifying enzymes alters HS structure and expression [63]. Structural studies suggest that HS can be modified at its principal FGF-binding sites and hence influences FGF activity in a tissue-specific manner [64].

HS promotes the binding of FGF to FGFR and stabilizes the signal-transducing dimer by enhancing protein–protein interactions at the dimer interface. Upon dimerization, the tyrosine transphosphorylation of intracellular kinase domains takes place, upregulating kinase activity, and leading to the formation of docking sites and phosphorylation of downstream signaling molecules. All of these signaling events ultimately affect signal transduction and the regulation of gene expression [65].

### Autocrine/Paracrine and Endocrine Signaling in Different FGF 

The effects of autocrine and paracrine FGFs are localized. HS can serve as a cofactor by mediating the binding between an FGF and FGFR during paracrine or autocrine FGF signaling. By contrast, a heparin-binding domain is absent in endocrine FGFs. Endocrine FGFs, as the name indicates, act as hormone-like signaling molecules; the absence of the HS-binding domain facilitates their respective functions [66]. These FGFs have a low affinity for HS, so they move away from cells, thus acting as hormones. Their receptor binding is mediated by Klotho proteins [67]. There are three main Klotho proteins: α-Klotho, β-Klotho, and γ-Klotho. α-Klotho and β-Klotho are crucial for the formation of endocrine FGFR complexes. Endocrine FGFs include FGF21, FGF15/19 (FGF15 in mice, FGF19 is its human ortholog), and FGF23. FGF21 is a starvation hormone that initiates a stress response by stimulating the sympathetic nervous system. FGF15/19 regulates the metabolic response to feeding and is a satiety hormone. It is involved in bile acid homeostasis and is considered to be a transversal metabolic coordinator [68]. FGF23, aside from being a phosphaturic hormone, serves as an important regulator of calcium and sodium reabsorption in the kidney [69]. Additionally, it functions in bone mineralization and is thought to regulate alkaline phosphatase expression. Usually, FGF23 is transmembrane-α-Klotho or soluble-α-Klotho dependent; however, bone mineralization is independent of α-Klotho [70]. Therefore, Klotho proteins are crucial for the regulation of metabolic processes in mammals [71]. 

## 4. FGF Mediation of Tissue Repair and Regeneration

Some of the major roles of FGFs are tissue repair and regeneration. In the FGF family, FGF2 (also known as bFGF) has been the protein of choice for improving wound healing in humans. FGF proteins are powerful mitogens that participate in both normal growth and wound healing. Among these proteins, bFGF is the most extensively studied one, with a recognized role in epithelial- and mesenchymal-cell proliferation as well as a putative function in angiogenesis [72]. Robson et al. have used bFGF, granulocyte-macrophage colony-stimulating factor (GM-CSF), or a placebo to treat 61 pressure ulcers; the ulcers treated with bFGF alone showed the best healing in terms of wound closure and contained higher levels of bFGF, PDGF, and TGF1 in the wound fluid [73]. Ohura et al. reported similar findings, claiming that the treatment of pressure ulcers with exogenous bFGF results in faster healing [74].

Acidic FGF, also known as FGF1, is a well-known and well-structurally characterized member of the FGF family, with structure, binding receptors, and physiological functions similar to those of bFGF. FGF1, FGF7, and FGF10 are three other FGF proteins that have a role in wound healing [75].

### 4.1. General Mechanisms of Tissue Repair and Regeneration

Tissue repair is a complex physiological process that involves multiple cell types, including keratinocytes, fibroblasts, endothelial cells, macrophages, and platelets [76]. It begins with the formation of a platelet plug to prevent blood loss. This process is a part of hemostasis. After fibrin matrix formation, the next step is inflammation, which protects the wounds from infection and removes debris. Neutrophils arrive at the site of damage in response to proinflammatory cytokines. If the induced immune response is insufficient, then the repair may be inefficient. After neutrophils, monocytes arrive and differentiate into macrophages. Given that macrophages are phagocytes, they clear debris and any neutrophils present at that site, a process known as efferocytosis. 

The next stage is proliferative, in which the wound gap is closed by keratinocytes and blood vessels are reformed via angiogenesis [77]. Fibroblasts deposit granulation tissue at the site of the initial fibrin clot; then, growth factors are released by platelets, endothelial cells, leukocytes, and fibroblasts [78]. These events not only attract immune cells and fibroblasts to the wound but also stimulate cellular proliferation and induce angiogenesis. Furthermore, they promote the secretion of cytokines and growth factors from surrounding cells. Growth factors participating in wound healing include granulocyte colony-stimulating factor (G-CSF), transforming growth factor (TGF), platelet-derived growth factor (PDGF), FGFs, vascular endothelial growth factor (VEGF), epidermal growth factor (EGF), keratinocyte growth factor (KGF), and GM-CSF. It has been demonstrated that growth factors stimulate collagen deposition and are involved in the secretion of collagenases, which degrade collagen during its remodeling [79]. Additionally, regulatory T cells are important for the final stages of wound healing. Re-epithelization proceeds in parallel to the above-mentioned phases; in response to the production of cytokines, epithelial cells migrate to the border of the wound to close it [80]. 

The last stage of tissue repair is remodeling (maturation), which is responsible for reshaping and reorganizing the deposited extracellular matrix to recover the structure of the respective normal tissue [81]. The remodeling process may take a few days to several years [82]. During the remodeling, the various types of cells that are involved in the earlier stages of wound healing disappear [83]. Initially deposited type III collagen is replaced by type I collagen [79]. Tight cross-linking between collagen fibers ensues, which increases the tensile strength of the scar [84]. The remodeling process may take a few days to several years [80].

### 4.2. Fibroblast Growth Factor in Regeneration

Organ regeneration is frequent in most invertebrate phylae, particularly in Cnidarians, unlike in vertebrates [85]. The earliest appearance of FGF has been observed in Cnidarians, the most ancient metazoans. Hydra belongs to phylum Cnidaria, and has a tubular body with a simple nervous system consisting of multiple subsets of neurons [86]. Hydra, in fact, possesses four putative FGF genes [87]. FGF and VEGF regulate the development of the nervous system and blood vascular system [88]. Another study reported that VEGF and FGF mediate the regeneration of hypostomes and tentacles in hydra [89]. In Hydra vulgaris, a particular pharmacologic inhibitor of FGFR inhibited head regeneration [89,90]. *Planaria* flatworms have been found to have an FGF homolog. In planarian *Dugesia japonica*, *fgf* gene-encoding Djfgf was identified, which is expressed in auricles in the head and pharynx. FGF expression was shown to be higher in the cells around the wound during the early stages of planarian regeneration after injury. In planaria, during head regeneration, there was a particularly high induction of FGF expression [91].

FGF signaling has been investigated in echinoderms as well. In the sea cucumber *Stichopus japonicus*, which lives in the coastal waters of Korea, Japan, China, and Russia [92], HS treatment with or without FGF2 enhances neural stem-cell proliferation in a dose-dependent manner at concentrations of 200–800 ng/mL without inducing apoptosis [93]. In the regenerating tissues of the sea star *Asterias rubens*, a heparin-binding polypeptide was discovered with a structure similar to that of mammalian FGF2 and the ability to induce proliferation in mammalian cells [94]. FGF signaling is essential for skeleton formation in regenerating arms of the brittle sea star *Amphiura filiformis* [95].

Lower vertebrates, such as fish and amphibians, have greater regenerative capacity than higher vertebrates [96]. The zebrafish (*Danio rerio*) and the urodele amphibian axolotl (*Ambystoma mexicanum*) are two excellent models for elucidating organ regeneration, especially the function of FGFs [90]. In 2000, Poss et al. demonstrated that a specific FGFR antagonist inhibits caudal fin regrowth in zebrafish. The suppression of the development of the blastema, i.e., the mass of actively growing, undifferentiated cells at the site of amputation, is responsible for this phenomenon [97].

The overexpression of a dominant-negative mutant of FGFR1 can have a similar effect. It was reported that FGF20 is required for zebrafish fin regeneration [98]. The regeneration of the spinal cord, liver, heart, lateral line neuromast hair cells, rod photoreceptor cells, and an extraocular muscle in the zebrafish also require FGF signaling [99,100]. It was shown that bone morphogenic protein (BMP), FGF, Wnt, Notch, Shh, and Nodal–TGF-β signaling cascades are essential for the appropriate development of a regenerated tail, according to experiments on small-molecule inhibitors and heat shock-inducible inhibitory proteins; these requirements are similar to those of early embryonic patterning [101]. An FGF inhibitor hinders the regeneration of the *Xenopus* tadpole tail, and increased production of FGF8 was registered in regenerating larval limbs of the African frog, *Xenopus*, and axolotl. In axolotls and newts, the application of FGF2, FGF8, and BMP7 to skin lesions causes ectopic development of the limbs rather than ordinary wound healing. Nerves are required for limb regeneration in axolotls. The FGF8 generated in the axolotl’s spinal ganglia was shown to promote the regeneration of limbs via long axons [102,103]. FGFs are important for organ regeneration in invertebrate and lower-vertebrate models.

### 4.3. The Role of FGFs in Tissue Repair

Higher vertebrates, unlike invertebrates and lower vertebrates, have almost no ability to regenerate organs, with rare exceptions, such as the regeneration of amputated digits in mice during early postnatal development and liver regeneration after partial resection. In contrast, higher vertebrates efficiently regenerate tissues after a mechanical injury, burn, chemical damage, and FGF signaling plays a key part in these processes. In mice, the complete deletion of *Fgf2* or simultaneous knockout of *Fgfr1* and *Fgfr2* in keratinocytes significantly delays wound healing [104]. 

In oligodendrocytes, the simultaneous deletion of *Fgfr1* and *Fgfr2* reduced the healing of demyelinated wounds in the mouse spinal cord. The deletion of *Fgfr2* in mouse urothelium was shown to prevent urothelial regeneration after cyclophosphamide-induced injury [105]. After a bleomycin-induced injury, FGF2 knockout mice had poor epithelial recovery in the lungs [106]. Another study showed that FGF2 also decreases bleomycin-induced lung fibrosis in mice with inducible FGF2 expression [107]. In adult sheep, an effect of FGF2 expression was observed when it was injected into defective knees in the form of a recombinant adeno-associated virus vector. These data support the notion that recombinant FGF2—when administered by an appropriate gene delivery method—has the potential to enhance osteochondral repair [108]. 

Recombinant FGFs induce tissue repair in animal models, particularly in mice and rats [109]. In this context, FGF1 or FGF2 hastens the healing of skin wounds, diabetic ulcers, spinal cord damage, and bone fractures. Recombinant FGF proteins or genetic constructs coding for them have been used in these studies [110,111,112,113]. Healing efficiency is improved by FGF mutants with higher activity and stability, as well as by FGF administration using slow-releasing gels. FGFs have been approved for clinical wound-healing use in China and Japan [114]. There are several recent extensive studies on the use of recombinant FGFs for tissue repair.

### 4.4. Endocrine FGFs in Repair Processes

The involvement of canonical (secreted HS proteoglycan–binding) FGFs in tissue repair has been extensively documented. Nonetheless, the roles of intracellular FGF11–14 and hormonelike FGF15/19, FGF21, and FGF23 in this process have received less attention. Intracellular FGFs can induce tissue repair. FGF11, a factor that is generated under hypoxic conditions, boosts in vitro angiogenesis and stimulates osteoclasts’ bone-resorbing activity [115]. Hypoxia-induced FGF11 also interacts with HIF1—the primary transcription factor involved in the hypoxia response—to increase its stability [116]. In addition, several studies have shown that hormone-like FGFs stimulate tissue repair. Knocking down the expression of their coreceptor Klotho delays cutaneous wound healing and strengthens the production of proinflammatory cytokines in a mouse model [117]. FGF21 increases functional recovery from a spinal cord injury by inhibiting injury-induced cell autophagy, implying that the systemic injection of FGF21 may be beneficial for spinal cord injury repair [118]. Finally, FGF15-deficient mice possess a significantly reduced ability to repair the liver after a partial resection [119].

### 4.5. Cell Processes Underlying the Stimulation of Tissue Repair by Fibroblast Growth Factor

**Figure 4:**
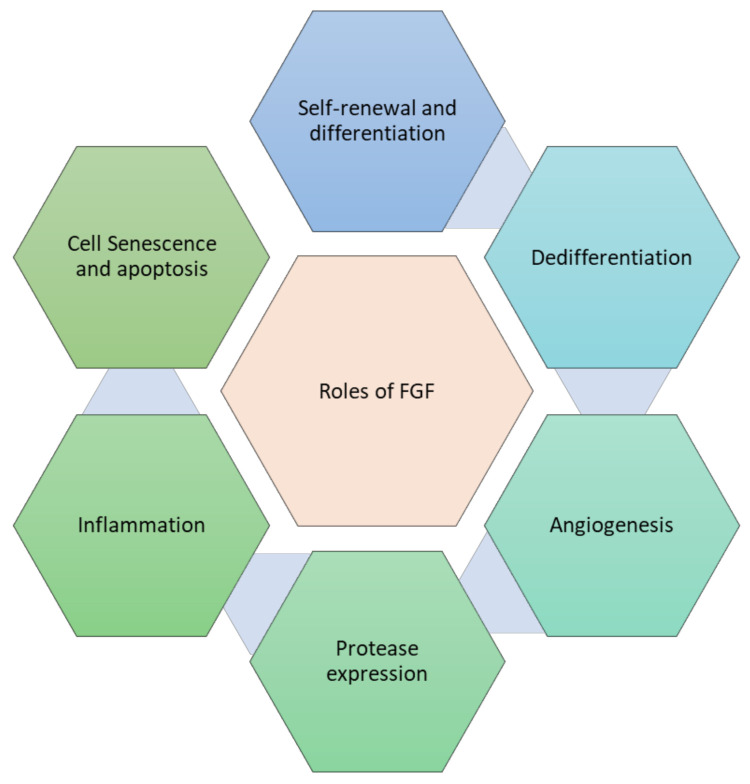
Role of FGF in tissue repair processes.

FGFs regulate various components of the cell phenotypic important for effective tissue repair, as published in numerous in vitro and in vivo experiments. The role of FGF in tissue repair is shown in Figure 4.

#### 4.5.1. Self-Renewal and De-Differentiation 

Tissue repair can be implemented via the partial dedifferentiation of differentiated cells, which increases their proliferation and migration. The cells are dedifferentiated to form pluripotent stem cells for the regeneration of damaged organs [120]. FGFs have long been known to effectively induce dedifferentiation. Kleiderman et al. recently showed that adding recombinant FGF2 to nonproliferating cultures of stem cell-derived murine astrocytes induces their proliferation and conversion to neurogenic stem cells [121]. Murota and colleagues have found that in a skin wound treated with recombinant FGF2, wound-edge keratinocytes undergo an enhanced epithelial–mesenchymal transition (EMT): a dedifferentiation process accompanied by the overexpression of EMT transcription factors like SNAI2, the decreased expression of epithelial marker E-cadherin, and the overexpression of EMT-specific transcription factors such as SNAI2. As a consequence, wound healing is expedited [122]. 

Saera-Vila et al. have reported that extraocular muscles of the zebrafish can be dedifferentiated by a myocyte dedifferentiation method. Nevertheless, when FGF signaling was inhibited, caspase 3 expression decreased, and cell proliferation decreased. These events point to the possibility that an FGF is involved in dedifferentiation. When MEK is inhibited by U0126, the effect is similar to that of FGF inhibition. In addition, ERK2 is upregulated when tissue regeneration begins after muscle injury. All this evidence suggests that FGFs partake in extraocular muscle regeneration [100]. 

In pathological conditions, FGF-induced cell dedifferentiation also occurs. Although FGFs cause vascular smooth muscle cells to dedifferentiate from a contractile to a synthetic phenotype in vitro, atherosclerotic plaques show stronger FGFR signaling and a lower expression of contractile proteins in smooth muscle cells. Chen et al. discovered that FGF and TGF signaling pathways have an antagonistic relationship in the mechanism controlling the smooth muscle cell phenotype [123]. In their study, FGF signaling induction inhibited TGF signaling and resulted in the synthetic phenotype in smooth muscle cells, whereas FGF signaling inhibition increased TGF signaling and yielded the contractile phenotype. Furthermore, the elimination of FRS2 in the context of the smooth muscle cell phenotype significantly reduced neointima development after carotid ligation. Although the smooth muscle cell phenotype and myofibroblasts have similarities, FGF2 inhibits the differentiation of fibroblasts into myofibroblasts [123]. 

#### 4.5.2. Effect on Proliferation 

In vitro and in vivo, HS proteoglycan-binding FGFs stimulate both proliferation and migration in a wide range of cell types. The presence of relevant FGFRs on a target cell’s surface is necessary for these actions. FGFs influence migration and proliferation through different mechanisms for the activation of different pathways. For instance, the launch of the migratory pathway is mediated by the SRC and p38 MAPK signaling cascades. On the other hand, if a pathway promoting proliferation is to be activated, ERK activation is necessary [108]. In a mouse model, FGF2 has been found to function in angiogenesis, migration, and proliferation through KDM2B-miR-101-EZH2 signal transduction [124].

Mammalian adult muscles have an excellent regenerative ability, with healing mediated by stem cells known as satellite cells, which are also required for physiological muscle growth. In mammals, these cells become activated after muscle damage, re-enter the cell cycle, and proliferate rapidly for a short period [125]. MiR-29a enhances the FGF2-induced proliferation of muscle progenitor cells and is a key target of FGFR signaling in muscle progenitor cells. Strategies that target miR-29a may be effective at maintaining muscle mass under certain conditions involving abnormal FGF2 signaling, e.g., during aging [126].

#### 4.5.3. Suppression of Cell Senescence and Apoptosis

Cellular senescence can be caused by internal and external factors. One of the main internal factors is telomere shortening, a critical determinant of cell lifespan that is involved in the aging process as well. Telomere shortening leads to the onset of DNA damage and hence cell senescence [127]. Telomerase is the enzyme responsible for maintaining telomere length and thus is crucial for the genetic stability of those cell types that undergo large numbers of divisions [128]. Conversely, cells that do not express telomerase cannot undergo an unlimited number of divisions; otherwise, they may become unstable. In this context, the p53, p21, and pRb pathways are activated, leading to growth arrest and cell senescence. The length of telomeres in laboratory mice is much greater than those in humans [129]. The human telomerase catalytic subunit (hTERT) regulates telomere homeostasis, genomic stability, cell proliferation, and tumorigenesis. It induces an FGF and downregulates p52, thereby inhibiting apoptosis [130]. FGF has been found to inhibit cellular senescence in mesenchymal stem cells. An FGF helps maintain the multipotency of cells and acts as a mitogen via ERK1/2 stimulation [131]. Zou et al. reported that FGF2 expression in human embryonic stem cell culture systems can be manipulated to create cells with longer telomeres, which may be beneficial for regenerative medicinal applications of human embryonic stem cells [132]. When human umbilical vein endothelial cells are grown with bFGF, they can attain a normal lifespan by upregulating telomeres [133]. 

Senescence and apoptosis are critical for the removal of precursor cells that do not follow a skeletogenic mechanism. Digit formation is an example of embryonic remodeling through cell degeneration, which proceeds via senescence and caspase-dependent apoptosis. Epigenetic regulators, Ubiquitin-like with PHD and RING Finger domain (UHRF) genes including *Uhrf1* and *Uhrf2*, are expressed in proapoptotic regions. UHRFs modulate cell differentiation, promote apoptosis, induce cell senescence, and are regulated by an FGF [134]. Page et al. reported that when FGF2 is added to a culture medium, it extends the in vitro lifespan of human fibroblasts and boosts the production of the stemness transcription factors OCT4, SOX2, and NANOG [135]. In bone-derived mesenchymal stem cells, FGF2 inhibits cellular senescence through the PI3K–AKT–MDM2 pathway, promoting cell proliferation [136]. In developing limbs, oxidative stress promotes the expression of BTG2, which induces cell-cycle arrest and caspase-mediated apoptosis, regulating senescence marker expression at the transcriptional level. Nevertheless, FGF2 and IGF1 downregulate *Btg2* [137]. Jung et al. have reported that HS, as a cofactor of FGFs, is crucial for preventing cell senescence. They suggested that FGFR1-mediated AKT signaling is important for premature senescence in an HS-dependent manner. Accordingly, inhibition of FGFR1 or AKT downregulates p53 and p21, causing cells to exit cellular senescence and enter an apoptotic state [138].

The effects of FGFs on apoptosis have been investigated for a long time. The inhibition of apoptosis was studied by Chow et al. in 1995, when they reported the suppression of apoptosis and induction of differentiation by an FGF in fiber cells of the mouse lens [139]. In 1996, FGF4 was found to hinder apoptosis in dental mesenchymal cells [140]. FGF2 has been shown to protect small-cell lung cancer cells from apoptosis by enhancing the expression of the anti-apoptotic proteins XIAP and Bcl-XL. This effect is mediated by the formation of a complex of B-Raf, PKCε, and S6K2. In this way, FGF2 induces chemoresistance in small-cell lung cancer cells [141]. In a study on non-small cell lung cancer, the inhibition of FGF signaling by FGF trapping and treatment with erdafitinib (a tyrosine kinase inhibitor) caused apoptosis in FGF-dependent human squamous cell carcinoma cells [142]. FGF2 has also been found to suppress apoptosis in neuronal cells by upregulating PI3K signal transduction. On the other hand, PD173074, a pan-FGFR inhibitor, attenuates the antiapoptotic effects of recombinant FGF2 by downregulating p-AKT and BCL2, thereby ultimately upregulating BAX [143]. In renal progenitor cells, an FGF is responsible for the inhibition of apoptosis caused by the loss of Wilms tumor repressor 1, *Wt1,* which is required for progenitor survival [144].

#### 4.5.4. Regulation of Inflammation

Inflammation induced by trauma is an integral part of the reparative process, in which both resident and invading inflammatory cells help regenerate the affected tissue. FGFs have long been known for their ability to control inflammation. FGF2 promotes proinflammatory cytokine production by primary human aortic smooth muscle cells and their conversion from a contractile to secretory phenotype, according to Qi and Xin [145]. The kinase activity of FGFR1 is required for the cytokine-induced activation of proinflammatory nuclear factor kappa B (NF-κB) signaling in hepatic stellate cells [146]. In prostate cancer cells, FGFR1 is also necessary for NF-κB signaling enhancement, which is dependent on TAK1 kinase stability [147]. TNF-α expression is promoted by FGF7 in immortalized human keratinocytes via the FGFR2–AKT–NF–κB signaling axis [148]. FGF2 increases the interleukin 1β (IL-1β)-dependent production of a pro-inflammatory protein called substance P and its receptor, NK1R, in human articular chondrocytes [149]. In this regard, it is worth noting that NF-κB signaling and IL-1β expression are both required for the formation of the FGF memory.

Inflammatory cells, such as T lymphocytes and macrophages, infiltrate tissues when stimulated by FGF2. After kidney ischemia-reperfusion injury, the transgenic overexpression of FGF1 in endothelial cells promotes macrophage infiltration [150]. Meij et al. have demonstrated that the transgenic overexpression of FGF2 in cardiomyocytes increases T-lymphocyte infiltration into the heart after treatment with isoproterenol [151].

Several research groups have reported anti-inflammatory effects of FGF1 in vivo, in contrast to the evidence of NFB-mediated proinflammatory effects of FGFs. The overexpression of FGF2 in the rat hippocampus using herpes simplex virus, for example, attenuates the IL-1β overexpression associated with experimental epileptogenesis [152]. In mice, the injection of FGF1 or FGF2 reduces inflammatory responses associated with acute pancreatitis [153]. FGF1 treatment diminishes both TNF-α and IL-6 release in a mouse model of diet-induced obesity; this phenomenon was attributed to a reduction in proinflammatory JNK signaling. It is possible that the effects of FGFs on inflammation are influenced by the dose and duration of recombinant FGF administration as well as by the tissue environment [154]. 

However, FGF23, known as phosphaturic hormone, is regulated by inflammatory cytokines and in turn upregulates the inflammatory cytokines. This vicious cycle, when activated, results in the uncontrolled production of inflammatory cytokines as well as FGF23. As a result of the production of these factors, tissue injury occurs. Along with the localized effect, distant tissues are also damaged [155]. It has been proposed that downregulation of TNF-α can help in reducing the level of FGF23 and hence cope with the consequences [156]. According to a study published by Yanucil et al., an FGFR4 blockade can be a safe alternative to FGF23 inhibition in order to deal with chronic inflammation in patients with chronic kidney disease [157]. Another study published by Grabner et al. suggested that FGF23 was not able to induce inflammatory effects in FGFR4 knockout mice and that the inflammatory effects of FGF23 were reduced by using an FGFR4-blocking antibody [158]. 

#### 4.5.5. Induction of Angiogenesis 

Angiogenesis is the formation of new blood vessels from the existing vasculature. Angiogenesis plays a major role in wound healing. The angiogenic response is needed to deliver immune cells, remove debris, and provide nutrients for tissue regeneration [159]. Vasculogenesis is the formation of new blood vessels from mesoderm-derived hemangioblasts [160]. FGFs are considered angiogenic factors and play a key role in neovascularization by mediating vascular assembly and sprouting [161]. In endothelial cells, the binding of FGF2 to FGFR1 initiates a proangiogenic complex program [162]. The function of FGFs as proangiogenic factors has already been established in vitro and in vivo. In epithelial cells, migration and capillary morphogenesis are stimulated upon the binding of an FGF to FGFR1 when epithelial cells are cultured on a collagen gel. Furthermore, this event launches the PI3K pathway [163]. In a mouse model, an FGF was found to be crucial for the development of coronary vessels. An FGF initiates the Hedgehog signaling cascade, which regulates VEGF signal transduction [164]. FGFs also interact with other growth factors and chemokines and facilitate the formation of blood vessels and arteries [165]. In corneal neovascularization, both acidic and basic FGFs are detectable in capillaries and corneal stromal cells. It has been suggested that anti-FGF and anti-FGFR antibodies can serve as good therapeutics against corneal neovascularization [166]. Because angiogenesis is a hallmark of cancer, FGFs are regarded as major therapeutic targets in cancer [167].

#### 4.5.6. FGF Impact on Protease Expression

Proteases are protein-degrading enzymes that are important for wound healing and tissue repair processes [168]. FGFs are essential for wound healing and tissue repair because they regulate multiple proteases by enhancing their expression, resulting in extracellular-matrix remodeling and promoting angiogenesis. *FGF2* gene introduction into HT-1376 cells upregulates matrix metalloproteinases (MMPs) 2 and 9. Antisense oligonucleotides against *FGF2* downregulate MMP2 in KoTCC-1 cells [169]. In hepatic stellate cells, an FGFR inhibitor alleviates apoptosis, inflammation signs, and MMP9 expression [170]. In another study, it was documented that the stimulation of FGFR1, but not FGFR2, leads to the production of proteases [161]. MMP1 and MMP13 are upregulated by FGF2 overexpression in human chondrocytes [171]. In a rat cell model of ethyl-N-nitrosourea-induced mammary adenocarcinoma, FGF1 promoted MMP9 expression by activating the PI3K–AKT pathway and increasing the DNA-binding affinity of NF-κB and AP-1 (i.e., activating protein 1) [172]. On the other hand, MMPs facilitate cancer cell metastasis. FGF2 enhances MMP9 activity through Ras stimulation, while MMP2 remains unaffected, in MCF-7 cells [173].

## 5. Conclusions

Increasing evidence suggests that FGF is involved in the regulation of the regeneration and repair process. In invertebrates and lower vertebrates, FGF is crucial for regeneration, whereas in higher vertebrates, it mediates the mechanisms of wound healing and tissue repair. FGFs stimulate tissue repair by maintaining pluripotency and help with self-renewal. They also stimulate proliferation and inhibit cell senescence and apoptosis. FGF induces angiogenesis and upregulates protease expression and helps in tissue repair and wound healing. 

China and Japan have focused on utilizing the potential of FGF for wound healing, particularly in the case of surgical wounds, including skin grafts, obstetric wounds, and surgical incisions, and for the treatment of burns and ulcers [174,175,176]. FGF1, FGF2, FGF4, FGF7, FGF16, FGF21, and FGF23 have been found to have good therapeutic outcomes for diabetic foot ulcers [177]. From the literature reviewed above, it seems that FGF can serve as a very promising therapy for tissue repair. There are no reports about the adverse effects of FGF on wounds and tissues. Further studies can be conducted to investigate adverse effects and clinical trials can be launched for investigating the efficacy of FGF in humans. 

## Figures and Tables

**Table 1:** FGF members and their distribution in subfamilies.

Subfamily	Members
FGF1 subfamily	FGF-1, FGF-2
FGF4 subfamily	FGF-4, FGF-5, FGF-6
FGF7 subfamily	FGF-3, FGF-7, FGF-10, FGF-22
FGF8 subfamily	FGF-8, FGF-17, FGF-18
FGF9 subfamily	FGF-9, FGF-16, FGF-20
FGF11 subfamily	FGF-11, FGF-12, FGF-13, FGF-14
FGF19 subfamily	FGF-19, FGF-21, FGF-23

**Table 2:** Interaction of FGF with their receptors.

Growth Factor	Interacting with the Receptor	References
FGF1	FGFR1 IIIb, FGFR1 IIIc, FGFR2 IIIb, FGFR2 IIIc, FGFR3 IIIb, FGFR3 IIIc, FGFR4	[37,38,39,40]
FGF2	FGFR1 IIIb, FGFR1 IIIc, FGFR2 IIIc, FGFR3 IIIc, FGFR4	[40,41,42,43]
FGF3	FGFR3	[44]
FGF4	FGFR2 IIIb, FGFR1 IIIc, FGFR2 IIIc, FGFR3 IIIc, FGFR4	[45,46]
FGF5	FGFR3 IIIc	[35]
FGF6	FGFR1 IIIc, FGFR2 IIIc, FGFR3 IIIc, FGFR4	[47]
FGF7	FGFR2 IIIb	[47]
FGF8a	FGFR1	[48]
FGF8b	FGFR2 IIIc, FGFR3 IIIc, FGFR4	[2]
FGF8c	FGFR3 IIIc, FGFR4	[2]
FGF8f	FGFR2 IIIc, FGFR3 IIIb, FGFR3 IIIc, FGFR4	[2]
FGF9	FGFR1 IIIb, FGFR2 IIIb, FGFR3	[38]
FGF10	FGFR2	[49]
FGF15	FGFR4	[50]
FGF16	FGFR1 IIIc, FGFR2 IIIc, FGFR3 IIIb, FGF3 IIIc	[47]
FGF17b	FGFR2IIIc, FGFR3IIIc, FGFR4	[2]
FGF18	FGFFR3	[51]
FGF19	FGFR4	[52]
FGF20	FGFR1	[53]
FGF21	FGFR1, FGFR2	[39]
FGF22	FGFR1 IIIb, FGFR2 IIIb	[47,54]
FGF23	FGFR1 IIIc, FGFR2 IIIc	[47,54]

## Abbreviations

AP-1	activating protein 1
bFGF	basic fibroblast growth factor (FGF2)
EGF	epidermal growth factor
ERK	extracellular signal-regulated kinase
FGF	fibroblast growth factor
G-CSF	granulocyte colony-stimulating factor
GM-CSF	granulocyte macrophage colony-stimulating factor
HS	heparan sulfate
IL-1β	interleukin 1β
IL-6	interleukin 6
JNK	c-Jun N-terminal kinase
KGF	keratinocyte growth factor
MAPK	mitogen-activated protein kinase
MMP	matrix metalloproteinase
NF-κB	nuclear factor kappa B
PDGF	platelet-derived growth factor
PI3K	phosphoinositol-3 kinase
SEF	similar expression to FGF
STAT	signal transducer and activator of transcription
TGF	transforming growth factor
TNF-α	tumor necrosis factor alpha
VEGF	vascular endothelial growth factor
